# Influence of the chemical content of sawdust on the levels of important macronutrients and ash composition in Pearl oyster mushroom (*Pleurotus ostreatus*)

**DOI:** 10.1371/journal.pone.0287532

**Published:** 2023-06-29

**Authors:** Kwadwo Boakye Boadu, Rosemary Nsiah-Asante, Richard Tuffuor Antwi, Kwasi Adu Obirikorang, Rogerson Anokye, Michael Ansong

**Affiliations:** 1 Faculty of Renewable Natural Resources, Department of Wood Science and Technology, Kwame Nkrumah University of Science and Technology, Kumasi, Ghana; 2 The Institute of Tropical Agriculture, Domeabra, Kumasi, Ghana; 3 Faculty of Renewable Natural Resources, Department of Fisheries and Watershed Management, Kwame Nkrumah University of Science and Technology, Kumasi, Ghana; 4 Faculty of Renewable Natural Resources, Department of Silviculture and Forest Management, Kwame Nkrumah University of Science and Technology, Kumasi, Ghana; ICAR-Directorate of Mushroom Research, INDIA

## Abstract

Influence of chemical composition of sawdust on the nutritional profile of oyster mushrooms (*Pleurotus ostreatus*) has yet to receive significant research attention. This information will help mushroom growers to select specific sawdust for the production of mushroom with desired dietary preferences. This study assessed the influence of the chemical composition of sawdust on the macronutrients and ash content of the pearl oyster mushrooms. The American Standard for Testing Materials and other widely accepted protocols were used to determine the C-N ratio, pH, lignin, hemicellulose and cellulose contents of mixed sawdust from tropical wood species. The study evaluated the fat, crude fibre, crude protein, carbohydrate, and ash content of the oyster mushroom cultivated on the sawdust. Cellulose constituted the largest component of the sawdust (47.82%), followed by lignin (33.29%). The yield of the mushroom (on 0.05 kg of sawdust) ranged from 490.1 to 540.9 g (biological efficiency: 44–50%); the average carbohydrates constituent in the mushroom was 56.28%. pH of the sawdust influenced the crude protein, carbohydrate, fat and ash content of oyster mushrooms (p<0.05) most significantly. The hemicelluloses also had a significant effect (p<0.05) on the mushroom’s minerals, fat and crude fiber content. The study revealed that the mushroom producers would likely obtain high protein content using sawdust with low pH (slightly acidic to slightly basic) in the oyster mushroom. Mushrooms grown on substrates, rich in hemicelluloses, had low fat and high crude fiber content.

## Introduction

The Pearl Oyster Mushroom (*Pleurotus ostreatus*) is the second most extensively grown and widely distributed edible mushroom in the world [1]. Oyster mushrooms grow quickly all-round the year in a wide range of temperatures and have a high level of pest and disease resistance [2,3]. Due to its macro- and micronutrient contents, oyster mushrooms are frequently utilized as dietary supplements [4–6]. Basidiocarp, the edible portion, of the mushroom has a low calorific value (1510 *kJ kg*^−1^), making it a suitable ingredient for diets with calorie restrictions [7]. The oyster mushroom has low-fat content, no cholesterol and total carbohydrate content of 26–82% [8]. These dietary benefits increase blood circulation, lowering the risk of anemia and heart diseases. Additionally, it is regarded as a good source of dietary fiber, which can assist in controlling energy intake and reducing obesity [9]. It is predicted that the global market for edible mushrooms, including oyster mushrooms, would increase from 50 billion in 2021 to 78.9 billion in 2026 [10]. The increase in consumers looking for environmentally friendly, reasonably priced, and cost-effective food sources is anticipated to fuel the growth [11]. Another element that is anticipated to support the expansion of the edible mushroom business is consumers’ growing propensity for vegetarianism [12]. The availability of suitable substrates, such as sawdust for mushroom growing, is one potential limiting factor for the growth of the worldwide mushroom trade. Providing appropriate substrates for mushroom cultivation is essential to the mushroom supply chain’s success.

The type and chemical characteristics of the substrates (such as pH and the C-N ratio) employed in mushroom cultivation impact the proximate composition and growth of the mushroom fungi [13]. As a result, different kinds of mushrooms thrive in diverse substrate types [5]. This knowledge is crucial for choosing appropriate substrates for growing various mushroom species. The influence of sawdust from several wood species, such as *Triplochiton scleraxylon*, *Ceiba petandra*, and *Terminalia superba*, on the growth and yield of oyster mushrooms has been thoroughly studied by previous authors [13–15]. However, there has been little research done on the direct effects of the different sawdust components on the macronutrients and minerals in oyster mushrooms. Hoa et al. [5] investigated the effect of C:N ration of the substrate on crude protein fraction of the mushroom for the first time. Later, the relationship between hemicellulose, cellulose, and lignin concentrations of substrates and sixteen elements (14 metals and 2 metalloids) in oyster and poplar mushrooms (*Cyclocybe cylindracea*) was also studied by Koutrotsios et al. [16]. They reported that the pH, P and K levels, as well as the substrates’ crude composition, had an impact on the concentrations of Mg, Cu, Cd, and Zn in both species.

There is currently a paucity of knowledge regarding how the ash, crude protein, crude fiber, carbohydrate, and fat contents of oyster mushrooms are affected by the C-N ratio, pH, lignin, hemicellulose, and cellulose contents of sawdust. The current study examined the chemical composition of sawdust and their effect on the macronutrients (i.e., crude fiber, crude protein, fat, and carbohydrate) and minerals in oyster mushrooms. The results of this study will help mushroom producers to choose specific sawdust with certain chemical qualities for the production of oyster mushrooms in order to satisfy the needs of people with dietary preferences for mushrooms.

## Materials and methods

### Collection and processing of sawdust

About 500g of mixed sawdust from three tropical woods [Kapok (*Ceiba pentandra)*, Teak (*Tectona grandis)* and Wawa (*Triplochiton scleroxylon*)] were obtained from timber processing firms at the Sokoban Wood enclave in Kumasi, Ghana (Google map coordinates: 6°37.264´N, 001°36.279´W). These species were chosen because of its ample availability and low cost. The sawdust was ground into a fine powder using a Wiley Mill (Model: Thomas T4274.E15), then air dried for a week at 25°C on corrugated paper. The ground sawdust was sieved to remove any particles larger than 0.5 mm and then used to examine the chemical composition.

### Determination of the chemical components of sawdust

#### The pH of the sawdust

The pH of the sawdust was determined by following the methods of Cavins et al. [17]. A portion of the grounded sawdust (2 g) was saturated with distilled water (40 cm^3^) for 24 h. The water leached from the sawdust was collected in a saucer. The pH of the leachate was measured immediately using a pH meter. The pH measurement was repeated 5 times to obtain the replicated value.

#### Preparation of extractive-free material for the determination of lignin, holocellulose and cellulose

The wood material was freed from extractives in accordance with the ASTM D 1105–96 standard [18] before the lignin, cellulose, and hemicellulose contents of the sawdust were determined at the Chemistry Laboratory of the Faculty of Renewable Natural Resources, Kwame Nkrumah University of Science and Technology (KNUST), Kumasi, Ghana. The chemical analysis of the extractive-free air-dried sawdust was repeated fifteen times to obtain replicated results.

#### Lignin content of sawdust

Lignin content of the sawdust was assayed following the method of ASTM D 1106–96 standard [19]. For the analysis, 15 ml of cold sulphuric acid (72%) was gradually added to 1g of air-dried, extractive-free sawdust with constant stirring. The mixture was continuously stirred for two hours at 20°C in a water bath. The mixture was then washed using 560 ml distilled water into 1liter Erlenmeyer flasks to reduce the sulphuric acid content to 3%. With the addition of boiling water, the mixture was cooked for 4 hours while maintaining a steady volume. The insoluble materials (lignin) were filtered, rinsed with 500 ml of hot water, and dried in an oven for two hours at 105°C or until a constant weight was achieved. 1 g of un-extracted sample was oven-dried to achieve a constant weight. The lignin percentage was calculated as follows:

$$Lignin\;(\%)=\frac{oven-dried\;weight\;of\;lignin}{oven-dried\;weight\;of\;un-extracted\;sample}\times 100$$ (1)

#### Holocellulose content of sawdust

Determination of holocellulose fraction of sawdust was done according to ASTM D 1104–96 standard [20]. Exactly 2 grams of air-dried extractive-free sawdust was added with a solution of sodium acetate (8.6 g), sodium chlorite (6.6 g), and ethanoic acid (5.7ml) in distilled water (180 ml) The mixture was incubated at 60°C in a water bath under a fuming chamber for 4 hours till the liquid and sample both turned pale and yellowish, respectively. The sample was filtered, cleaned with distilled water, and dried in an oven for five hours at 105°C. Following oven drying, the leftover was weighed accurately. A 2 g unextracted sample of sawdust was dried in an oven at 105°C until constant weight. The holocellulose content was obtained following the formulae:

$$Holocellulose\;(\%)=\frac{x}{y}\times 100$$ (2)

Where: x = Oven-dry weight of the residue; y = Oven-dry weight of the un-extracted sample.

#### Cellulose content of sawdust

Cellulose fraction of sawdust was determined following the method of ASTM D 1103–60 [21]. The sawdust sample (2 g) was dried in the oven, weighed, and then put into 250 ml beakers with glass covers. The sample was subjected to a 45-minute treatment with 25 ml of 17.5% NaOH. For the treatment, 10 ml of the NaOH solution was well mixed with the sample and swirled with a glass rod before being placed in a water bath at 20°C. Another 5 ml of the 17.5% NaOH was added and gently stirred five minutes later. The remaining 10 ml of NaOH was painstakingly stirred into the mixture and added in two portions at 5-minute intervals. Later, 33 ml of distilled water (20°C) was thoroughly mixed with the mixture and incubated for one hour at 20°C. The solution was filtered and the residue was successively washed with 100 ml of 8.3% NaOH and distilled water. The residue was treated with 15% acetic acid at 10% strength for 3 minutes and then thoroughly rinsed with distilled water. Finally, the residue was dried at 103 ± 2°C until a constant weight was attained. The cellulose content was calculated to be:

$$Cellulose\;(\%)=\frac{w1}{w2}\times 100$$ (3)

*w*1 = weight of oven-dried residue; *w*2 = weight of original oven-dried wood sample.

#### Hemicellulose content of sawdust

Holocellulose is made up of soluble hemicellulose and insoluble cellulose, as determined by solubility in the 17.5% NaOH solution used to calculate the cellulose content. The difference between the holocellulose and cellulose contents of the wood sample was used to calculate the hemicellulose content [19]. The amount of hemicellulose was calculated as follows:

Hemicellulose(%)=H−C (4)

*H* = Holocellulose content; *C* = Cellulose content.

#### Carbon content of sawdust

The percentage of organic carbon in the sawdust was determined following the Walkley–Black Wet Oxidation method [22]. The sawdust (0.5 g) was put into an Erlenmeyer flask (500 ml) and about 10 ml of 1.0 N Potassium dichromate (K_2_Cr_2_O_7_) solution was added. The flask was swirled gently and about 20 ml of concentrated H_2_SO_4_ was added with a fast-flowing pipette. The flask was immediately whirled to allow the solution to touch the sawdust particles. The flask and the suspension were cooled for 30 mins on an asbestos sheet in a fume chamber. Two reagent blank solutions (without sawdust) were prepared to standardize the FeSO_4_ solution. 2.0 ml of diphenylamine indicator and 10 ml of conc. orthophosphoric acid were added and diluted to 200 ml with distilled water. The solution was titrated against 0.5 N FeSO4 solution till the violet blue color changed to dark blue and green. The titer value for the blank solution (≥ 10.5) was observed and updated. The formula used to determine the carbon percentage (C%) was:

$$C\;(\%)=\frac{M\times (Vbl-Vs)\times 0.003\times 1.33\times 100}{g}$$ (5)

M = FeSO_4_ Molarity; *V*_*bl*_ = Volume of FeSO_4_ used in blank titration; *V*_*s*_ = Volume of FeSO_4_ used in sample titration; *g* = mass of sawdust in gram; 0.003 = milli-equivalent weight of Carbon in grams (12/4000); 1.33 = correction factor.

#### Nitrogen content of sawdust

The nitrogen content of the sawdust was determined using the Kjeldahl method [23]. A Kjeldahl flask was filled with 10 ml of distilled water and about 1g of oven-dried sawdust. The mixture was given 10 minutes to moisten. The Kjeldahl catalyst, which contained one-part selenium, ten parts CuSO_4_, and one hundred parts Na_2_SO_4_, was applied using a spatula. Conc. H_2_SO_4_ (10 ml) was then added. The mixture was slowly heated until it turned a bright green color. The digest was added to a 50 ml flask once the flask had cooled. The digestion flask was rinsed with distilled water and diluted to 50 ml. 10 ml of the digest is added with 20 ml of 40% NaOH and 90 ml of distilled water. The solution was distilled and the distillate was collected in 10 ml 4% boric acid solution and three drops of mixed indicator (methyl orange + bromocresol green) in a 500 ml flask for five minutes. As a result of the addition of nitrogen, the distillate’s hue altered to a light blue. The distillate was titrated with 0.l N HCl until the blue hue abruptly flared to pink and then reverted to grey. The nitrogen content was determined using the formulae:

$$N\;(\%)=\frac{(a-b)\times 1.4\times N\times V}{S\times t}x\;100$$ (6)

Where: *a* = volume of Hydrochloric acid used in the sample titration; *b* = volume of Hydrochloric acid involved in the blank titration; *N* = HCl Normality; *V* = digest total volume; *S* = weight of oven-dried sample used for digestion; *t* = volume of aliquot used for distillation (10ml).

The nitrogen and carbon contents were used to determine the C-N ratio of the sawdust.

#### Ash content of the sawdust

The ash content of sawdust was determined according to ASTM D 1102–84 [24]. The quantity of ground sawdust added to the crucible was about 2 g. The sample was burned off by heating the crucible and its contents to 600°C for two hours in a muffle furnace. The final ignition occurred at a temperature of between 580 and 600°C. The ash-filled crucible was weighed after cooling in a desiccator. The ash content was determined as:

$$Ash\;content\;(\%)=\frac{w2-w1}{w3-w1}\times 100$$ (7)

*w*1 = empty crucible weight; *w*2 = weight of crucible containing ash; *w*3 = weight of crucible with sawdust sample.

### Preparation of compost and cultivation of Pearl oyster mushroom

**Fig 1:**
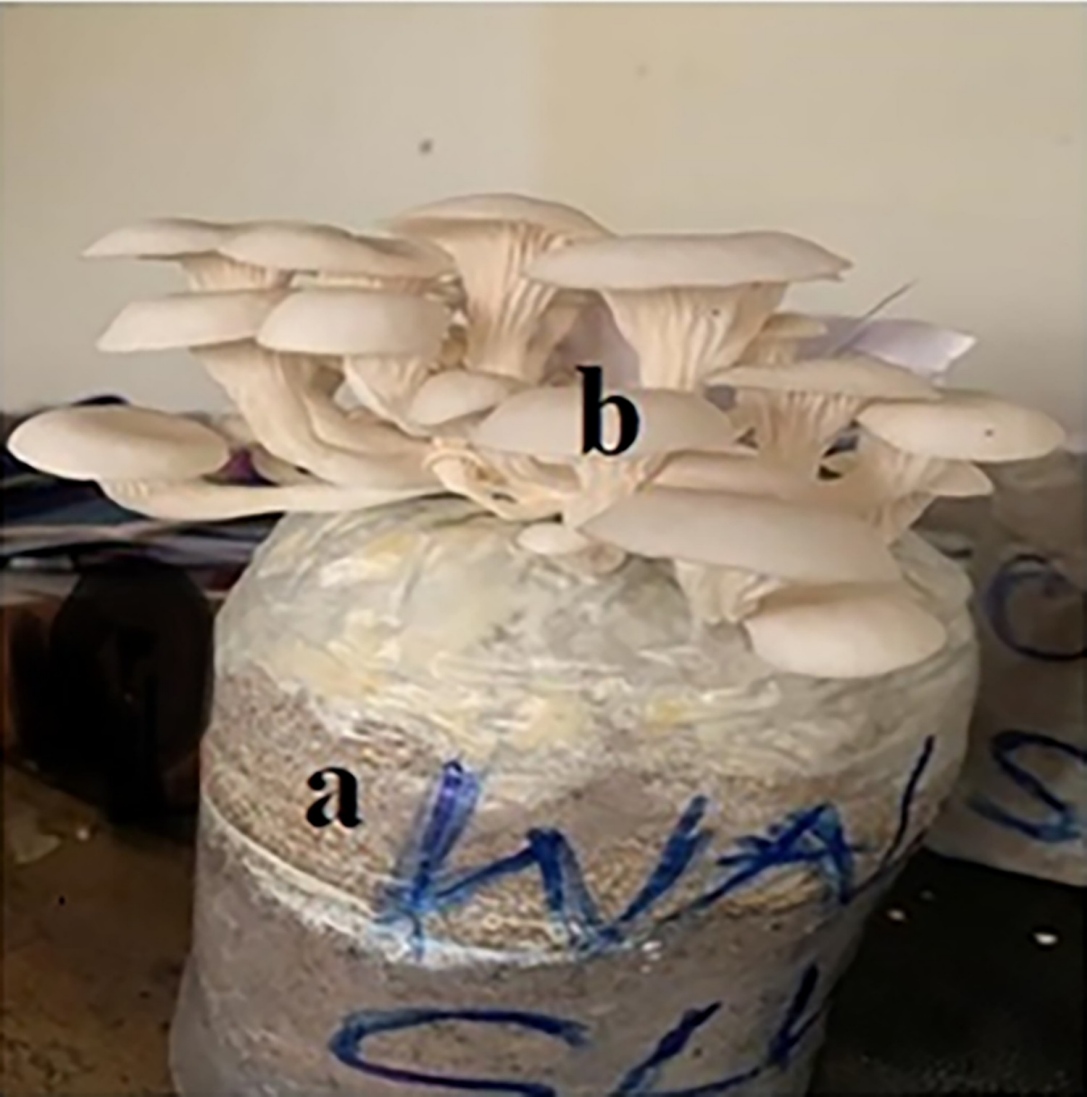
Sawdust substrate in plastic bag (a) with fruiting body of mushroom (b).

The preparation of compost and cultivation of Pearl oyster mushroom was done at the Mushroom Culture House of the Kumasi Institute of Tropical Agriculture (KITA) at Domeabra, Kumasi- Ghana (Coordinates: 6.67620, -1.50267). The standard methods described by Badu et al. [15] and Atikpo et al. [25] were used in composting sawdust and cultivation of oyster mushroom. After 10–15 days of fruiting, the oyster mushrooms (Fig 1) were harvested to determine their yield, macronutrients and mineral content. The experiment was replicated 15 times.

### Determination of the nutritional composition of mushroom

#### Yield and moisture content

The total weight (in grams) of fresh mushrooms harvested at the first, second, and third flush stages was added to determine the overall yield of the mushrooms. The moisture content of oyster mushrooms was assessed using the AOAC technique [26] at the Laboratory of the Department of Food Science and Technology, KNUST.

#### Fat content

Fat content of oyster mushrooms (on dry weight basis) was assessed in accordance with the procedures outlined by Masamba and Kazombo-Mwalo [27] using a Soxhlet extraction equipment. Following formula was used to determine the mushroom’s fat content:

$$\%\;fat\;(dry\;basis)=\frac{(weight\;of\;flask+fat)-weight\;of\;flask}{weight\;of\;sample}\times 100$$ (8)

#### Crude protein

The percentage of crude protein in oyster mushrooms was determined based on the Kjeldahl method according to Masamba and Kazombo-Mwalo [27] using 0.25 g of grounded mushroom, 98% sulphuric acid, and 0.01 M HCl. The crude protein content of the mushroom was calculated:

$$Crude\;protein\;(\%)=\frac{(N\times 14.007\times (Vs-Vb)\times 4.38)\times 100}{W\times 1000}$$ (9)

*N* = Standard HCl normality (0.01); *Vs* = volume of HCl employed for the titration; *Vb =* volume of blank solution; *W* = weight (g) of dry mushroom sample used.

#### Ash content

The ASTM D 1102–84 standard method [24] earlier described was used to determine the ash content of oyster mushrooms.

#### Crude fiber content

The method outlined by Alam et al. [28] was used to calculate the oyster mushroom’s crude fiber content. Approximately 5g of moisture and a fat-free mushroom sample were obtained using the Soxhlet extraction method. Mushroom’s crude fiber content was determined following the fomulae:

Crudefiber=100−(moisture+fat)×we−waweightofsample (10)

*we* = weight of oven-dried residue; *wa* = weight of empty petri dish

#### Carbohydrate content

The carbohydrate content of the mushroom was determined from the formula [28]:

Carbohydrate=100−(m+f+cp+a+cf) (11)

*m* = oyster mushroom moisture content; *f* = oyster mushroom fat content; *cp* = crude protein of oyster mushroom; *a* = mineral content of oyster mushroom; *cf* = crude fiber of oyster mushroom

### Data presentation and analysis

A bivariate Pearson correlation test was performed in SPSS (version 20; IBM, New York, United States) to assess the linear correlation among the independent variables and between the dependent and independent variables. The independent variables with significant correlation (p<0.05) with the dependent variables were put into a stepwise multiple regression model to determine the most significant predictive independent variables for each dependent variable. A preliminary analysis was done to ensure that the assumptions of normality, linearity, homoscedasticity and multicollinearity were not violated. The model’s goodness of fit was tested using the *R*^*2*^, standard error of the estimate and *F*-ratio in the ANOVA table.

## Results

### The chemical components of the sawdust and proximate composition of oyster mushroom

Table 1 lists the chemical components of sawdust. Cellulose was the main constituent of sawdust followed by Lignin. The pH of the sawdust was mildly basic and acidic (Table 1).

**Table 1:** Chemical properties of sawdust used for the production of oyster mushroom.

Chemical properties	N	Minimum	Maximum	Mean/Range	Std. Error	Std. Deviation
Cellulose (%)	15	47.03	48.88	47.82	0.14515	0.56216
Hemicellulose (%)	15	14.98	18.61	16.63	0.36919	1.42987
Lignin (%)	15	27.60	39.53	33.29	1.13504	4.39600
Ash (%)	15	1.30	3.44	2.26	0.18491	0.71617
pH	15	6.00	900	6–9	0.29449	1.14055
C-N ratio	15	72.51	85.50	76.68	0.95931	3.71540

The composition of mushroom grown showed carbohydrate component to be the maximum (56.28%) (Table 2). Average crude protein was 18.36%. The mushroom’s fat level was the lowest among its nutritious qualities, aside from its moisture content. The mushroom produced between 490.1 g and 540.9 g of yield on a 0.05 kg substrate.

**Table 2:** Proximate components of oyster mushroom.

Yield and nutritional composition of mushroom	N	Minimum	Maximum	Mean	Std. Error	Std. Deviation
Crude protein (%)	15	16.22	20.67	18.36	0.41669	1.61383
Carbohydrate (%)	15	53.29	58.71	56.28	0.49157	1.90383
Crude fibre (%)	15	7.82	11.28	9.09	0.31997	1.23923
Fat (%)	15	2.34	6.43	4.25	0.33097	1.28186
Ash (%)	15	6.67	9.80	8.17	0.25687	0.99484
Moisture content (%)	15	3.26	4.39	3.84	0.09727	0.37672
Yield (g)	15	490.1	540.9	515.5	10.231	40.10023

### Correlation between the chemical properties of sawdust and nutritional components of mushroom

The correlation studies showed that the C-N ratio and pH of sawdust’s were closely associated to each other. Also, a significant correlation was found between the hemicellulose, ash, and lignin (Table 3). Significant positive correlation was observed between hemicelluloses and lignin while strong negative correlation was found between hemicelluloses and ash, and lignin and ash. No significant correlation was observed between the sawdust’s cellulose with hemicellulose and ash contents

**Table 3:** Correlation matrix of the chemical properties of sawdust.

			
	Cellulose	Hemicellulose	Lignin	Ash	pH	C-N ratio
Cellulose	1.000					
Hemicellulose	0.253	1.000				
Lignin	0.151	0.883*	1.000			
Ash	-0.320	-0.960*	-0.897*	1.000		
pH	-0.028	0.256	0.643	-0.284	1.000	
C-N ratio	-0.238	0.087	0.433	-0.183	0.675*	1.000

*Correlation is statistically significant (p<0.05).

Crude protein and carbohydrate (p>0.05) of mushroom was not found correlated with sawdust cellulose content, however hemicelluloses of sawdust showed strong positive correlation with crude fibre of sawdust while strongly negatively correlated with crude fat of mushroom (Table 4). The chemical components of the sawdust, with the exception of pH, did not significantly correlate with the mushroom’s ash concentration. The lignin content of the sawdust negatively correlated with crude protein and crude fiber of the mushroom. It however had a positive correlation with carbohydrate and fat. The sawdust ash had a strong negative correlation with crude fiber and a strong positive correlation with the fat in the mushrooms.

**Table 4:** Correlation matrix of chemical properties of sawdust and nutritional components of oyster mushroom.

Chemical properties of sawdust	Nutritional components of mushroom				
	Crude protein	Carbohydrate	Crude fiber	Fat	Ash content
Cellulose	0.095	-0.147	0.400	-0.379	0.176
Hemicellulose	-0.307	0.131	0.949*	-0.838*	0.490
Lignin	-0.667*	0.482*	-0.816*	0.536*	0.132
Ash	0.336	-0.137	-0.939*	0.786*	-0.450
pH	-0.963*	0.917*	0.11	0.244	0.608*
C-N ratio	-0.654*	0.655*	0.016	0.273	-0.517

*Correlation is statistically significant (p<0.05).

### The influence of the components of the sawdust on the nutritional properties of Pearl oyster mushroom

The independent variables significantly predicted the mushroom’s crude protein [*F*(1, 13) = 166.490, *p* = 0.000, *R*^*2*^ = 0.928] (Table 5), carbohydrate [*F*(1, 13) = 68.616, *p* = 0.000, *R*^*2*^ = 0.841] (Table 6), ash content [*F*(2, 12) = 26.532, *p* = 0.000, *R*^*2*^ = 0.816] (Table 7), fat [*F*(2, 12) = 76.604, *p* = 0.000, *R*^*2*^ = 0.927] (Table 8) and crude fiber [*F*(1, 13) = 116.794, *p* = 0.000, *R*^*2*^ = 0.900] (Table 9).

**Table 5:** Multiple regression analysis of independent factors influencing the crude protein content of oyster mushroom.

Variable	Coefficient	Standard Error	t-statistic	p
Constant	29.008	0.833	34.820	0.000
pH	-1.363	0.106	-12.903	0.000

**Table 6:** Multiple regression analysis of independent factors influencing the carbohydrate content of oyster mushroom.

Variable	Coefficient	Standard Error	t-statistic	P
Constant	44.324	1.457	30.412	0.000
pH	1.547	0.185	8.283	0.000

**Table 7:** Regression analysis of independent factors influencing the ash content of oyster mushroom.

Variable	Coefficient	Standard Error	t-statistic	p
Constant	5.519	1.522	3.627	0.003
Hemicellulose	0.481	0.112	-6.118	0.000
pH	-0.684	0.089	5.388	0.000

**Table 8:** Multiple regression analysis of independent factors influencing the fat content of oyster mushroom.

Variable	Coefficient	Standard Error	t-statistic	p
Constant	14.311	1.231	11.629	0.000
Hemicellulose	-0.864	0.072	-11.972	0.000
pH	0.552	0.090	6.101	0.000

**Table 9:** Multiple regression analysis of independent factors influencing the crude fiber content of oyster mushroom.

Variable	Coefficient	Standard Error	t-statistic	p
Constant	-4.588	1.270	-3.614	0.003
Hemicellulose	0.822	0.076	10.807	0.000

The only independent variable that statistically significantly predicted or explained the variation in crude protein, carbohydrate, ash, and fat in the mushrooms was the pH of the sawdust. When all other factors were held constant, a one-unit increase in sawdust pH will cause a 1.363 unit decrease in crude protein, a 1.547 unit increase in carbohydrate content, a 0.684 unit decrease in ash content, and a 0.552 unit increase in fat content of the mushroom (Tables 5–8).

The ash, fat, and crude fiber of oyster mushrooms were likewise considerably influenced by the hemicellulose content of sawdust (Tables 8 and 9). When all other factors remained constant, a one-unit increase in hemicellulose will result in an increase of 0.481 and 0.822 units in the ash and crude fiber contents of the mushrooms, respectively. In addition, a one-unit increase in hemicellulose will cause a 0.864 unit drop in fat content of mushrooms.

## Discussion

### The chemical components of the sawdust

The cellulose and hemicellulose content of the sawdust used in the current study was comparable to that of the substrates suggested for the best growth of oyster mushrooms. The two main sources of carbohydrates needed for mushroom development are cellulose and hemicellulose [14,29]. Due to their high cellulose and hemicellulose contents (35–46%), it has been discovered that sugarcane bagasse, soy bean husks, corn stalks, and sawdust from three tropical wood species (*Triplochiton scleroxylon*, *Ceiba pentandra*, *and Terminalia superba*) all enhance the early growth of mushrooms [15,30]. The substrate’s lignin concentration (33.29%) in the employed sawdust further supports its suitability as a substrate for the growth of mushrooms. A low quantity of lignin in the growing substrate (~40%) is advised for the early growth of mushrooms because lignin encrusts cellulose and hemicellulose and decreases their accessibility to cellulolytic enzymes in mushrooms [29,31]. According to Osunde et al. [29], the lower C-N ratio of corncob (120:1) compared to sawdust from an unidentified plant (325:1) led to the better growth and yield of mushrooms grown on the former. As amino acids serve as the building blocks of proteins for mycelial growth and development, a low C-N ratio indicates that nitrogen is readily available for the biosynthesis of purines and pyrimidines [32]. In our investigation, the substrate’s C-N ratio (76.68:1) was lower than previously reported ratios. Oyster mushrooms grew and developed more quickly as a result of this. Oyster mushrooms with high protein content could potentially be produced due to the substrate’s low C-N ratio [25,29]. Sultana et al. [33] discovered that oyster mushrooms prefer a slightly acidic to a slightly basic substrate (pH range: 5–9). Mycelial development and subsequent fruiting body production are facilitated at a pH range of slightly acidic to slightly basic. Mushroom growth is hampered by low pH (very acidic) levels of <5. H^+^ is released in exchange for nitrogen when mushroom mycelium absorbs it from the substrate [33]. This can cause the pH to decrease and move closer to the acidic range. Therefore, Ghareeb [34] reported that the optimal substrate is neutral to slightly basic. This hypothesis is supported during the present study by the pH range (6–9) of the substrate for oyster mushroom development. In this investigation, the sawdust’s ash concentration was 2.26%. Wood ash concentration was found to be 1.49% by Miskam et al. [35], but Smoka-Danielowska [36] discovered that it fluctuates between 2 and 18.3%. According to Sales-Campos [37], the ash in substrates typically contains minerals including Ca, Cu, Fe, K, Mg, Mn, P, and Zn that may affect the mineral composition of mushrooms. During fruiting, the minerals are absorbed by the mushroom mycelium. As a result, these minerals are widely available for uptake by the mushroom due to the high ash content of the sawdust. Despite falling within the range suggested by Smoka-Danielowska [36], the study’s reported lower ash content than that of other substrates utilized for mushroom production, such as wheat straw (8.6%) and rice straw (14.65%) [38,39].

### The yield and nutritional properties of oyster mushrooms cultivated

The oyster mushroom’s overall yield (490.1–540.9 g) compares favorably to the results of Yang et al. [40] and Ejigu et al. [41]. Compared to the value typically reported for edible mushrooms by Sharma et al. [42] (22.89% and 29.97%) and Ashraf et al. [43] (19.83–24.23%), the protein content of Pearl oyster mushrooms found in this study (18.36%) was lower. During the study, carbohydrate content of oyster mushroom was 56.28 percent, which is consistent with Obodai and Apertorgbor’s findings [44]. The amount of carbohydrates in oyster mushrooms was observed to range between 45 and 65% by Badu et al. [15] and between 30.24 and 42.26% by Sharma et al. [42]. The difference in the reported values could be attributed to the difference in the cultivation media. About 2.5 g of dietary fiber is reported in a 100 g of mushrooms. During the present study, crude fiber content (9.09%) was within the range (6 to 14%) suggested by Sharma et al. [42]. The result for fat content obtained during the study was 4.25% and is also reported to be less than 6% [15,39]. The ash that remains after complete burning dry mushrooms is characterized by the mineral composition of mushrooms. Oyster mushrooms were shown to be rich in minerals by Yamauchi et al. [6], with potassium being the most prevalent mineral. According to Yamauchi et al. [6] and Alam et al. [28], the ash concentration of oyster mushrooms ranges from 6.3 to 9.02% while as per Badu et al. [15], the ash level of oyster mushrooms can be as low as 4.40%. Bhattacharjya et al. [45] reported this value from 8.5 to 13.0%. During the current study, the ash content of pearl oyster mushrooms was found to be 8.17%, which is consistent with the results obtained by Alam et al. [28] and Yamauchi et al. [6].

### The influence of the constituents of sawdust on the nutritional properties of oyster mushroom

The main factor that had a significant impact on the crude protein, carbohydrate, ash, and fat content of oyster mushrooms was the pH of the sawdust. The pH of the sawdust was inversely correlated with the crude protein and ash content of the mushrooms. There are several reports giving ideal pH range 5–9 for mushroom development [33,46]. We have also found that growth and development of oyster mushroom is facilitated by a pH range from slightly acidic to slightly basic. The precise reasons for mushrooms grown in pH ranges from slightly acidic to slightly basic have higher protein and ash levels are still mostly unknown. The best pH ranges for nutrient availability for absorption in various growth substrates are moderately acidic and neutral [47,48]. Certain nutrients may create insoluble compounds and become inaccessible for mushroom uptake when the pH is not ideal [47]. Additionally, it is possible to infer that the influence of pH on the substrate microbiota impacts the macronutrients and ash in mushrooms since some macrofungi cannot develop fruiting bodies in sterile settings [49]. Additionally, it has been noted that near-neutral pH levels can increase substrate microbial activity [50], and growing mushrooms in microbe-rich substrates might lead to positive interactions that enhance nutrient uptake [51]. For instance, the fungus *Agaricus bisporus* produces a system of bacteriolytic enzymes that serves as a nutrient-releasing system to gain additional nitrogen and provides antibacterial benefits to safeguard mycelia [52]. The higher protein accumulation in mushrooms can be explained by the better mycelia health and increased nitrogen availability. Some macrofungi may eat some symbiotic bacteria and digest them as sources of carbon and nitrogen [53]. This may help to partially explain the significant correlation between the protein content of pearl mushrooms and pH levels. Similar findings were obtained between the ash/mineral content of *P*. *ostreatus* and *C*. *cylindracea* and the substrate pH by Koutrotsios et al. [16]. Contrarily, the levels of carbohydrate and fat in this study rose linearly as pH rose. The observed pH for increased cellulase activity is within acidic ranges, despite the fact that pH is crucial for the breakdown of cellulose and the uptake of carbon by mycelia [54,55]. Thus, rather than carbon being readily available for consumption, the increasing carbohydrate levels with increasing pH in this study appear to inversely reflect the decreasing protein contents with increasing pH. When one macronutrient intake is increased, other important nutrient components usually decrease as well. According to Wilcox and Shibles [56], increasing the crude protein level of soybeans depletes its supply of carbs, and vice versa. According to Sultana et al. [33], oyster mushrooms produce more carbohydrates when the pH of the substrate is higher. The energy required for the production of fatty acids is known to be provided by carbohydrates in mushroom fruiting bodies. Therefore, an increase in carbohydrate content is likely to result in an increase in the mushroom’s fat content. When oyster mushroom producers employ sawdust with a pH range of slightly acidic to slightly basic, they are likely to succeed in their quest for high mushroom protein content.

The molecule in the sawdust that has the biggest influence on the amount of fat and ash in the mushroom is hemicellulose. According to Table 8’s negative hemicellulose coefficient, the amount of fatty acids in mushrooms tends to decrease as the substrate’s hemicellulose content rises. Additionally, Abou Fayssal et al. [57] reported a negative correlation between the fatty acid in mushrooms and the carbohydrates in a substrate made entirely of wheat straw. The activities of enzymatic degraders are stimulated by relatively low hemicellulose content, according to Koutrotsios et al. [4]. They found a negative correlation between substrate hemicellulose content and the biological effectiveness of mushrooms.

This study’s findings suggest that low hemicellulose substrate should be chosen for mushroom growing in order to produce mushrooms with a high level of fat. For the ash and minerals in mushrooms, the opposite is true. According to Koutrotsios et al. [16], *P*. *ostreatus* had higher concentrations of Cu, Fe, Mn, and Li when the proportion of hemicellulose in the substrates was reduced.

The independent variable that made a statistically significant difference in the prediction of mushroom crude fiber was hemicellulose. Crude fiber mostly consists of cellulose, lignin, hemicelluloses, pentosans, and trace amounts of crude protein and ash. Therefore, and the significant effect of hemicellulose on crude fiber was expected [58]. Different ratios of these components can be found in the crude fibre of mushrooms. According to Philippoussis et al. [14], hemicellulose shows a high linear association with the amount of mushroom fibre among the chemical constituents of the substrate. This explains the statistical importance of hemicellulose’s effect on the crude fibre of mushrooms. Therefore, oyster mushroom growers should utilize substrates with high hemicellulose to cultivate mushrooms with decent dietary fibre. This will result in the production of mushrooms that are high in dietary fibre.

## Conclusion

The current study looked at the different constituents in the sawdust and their effect on the macronutrients (such as crude fiber, crude protein, fat, and carbohydrate) and ash level in Pearl oyster mushrooms. The most important factor that adversely affected the crude protein and ash contents of oyster mushrooms and favorably affected their fat and carbohydrate contents was the pH of the sawdust. The levels of ash and crude fiber in the mushrooms significantly increased and its fat content decreased as the sawdust’s hemicellulose concentration rose. As a result, oyster mushroom farmers that aim for high protein and ash content in their mushrooms are likely to succeed when they utilize a substrate with a low pH (slightly acidic to slightly basic). Similarly, substrates rich in hemicellulose should be chosen for Pearl oyster mushroom production in order to produce mushrooms with low fat content and a high percentage of crude fiber.

## Limitation of the study

The mushroom’s enzymatic activities weren’t specifically looked into. However, the study’s findings and conclusions with regard to the work’s aims were unaffected by this constraint.
